# Researches on Digestion

**Published:** 1843-05

**Authors:** 


*Researches on Digestion.*—By Bouchardat and Sandras. By feeding dogs with pure fibre, killing them at different intervals after taking the food, and examining carefully the contents of the stomach, intestines, and the chyle itself, and comparing them with the appearances presented in dogs killed while fasting, Bouchardat and Sandras have arrived at results which, while opposed to the usual belief of the physiology of digestion, seem to bear out the conclusions drawn by the illustrious experimentalists. They found that the fibrin was converted into a fluid in the stomach, was in fact dissolved. They found that the fluid did not pass into the intestines, or but a very small portion only, that all the truly dissolved matters were removed like other fluids from the stomach itself. That the matters which were found in the intestines of the animal killed when fasting were identically the same as those of the animal fed with a full meal of pure fibrin, only the latter contained a slightly greater proportion of fibrin in a dissolved or fluid state. The chyle, too, of the fasting animal presented the exact same qualities as that of the animal which had a full meal of fibrin, the proportion of dissolved fibrin in it was only very slightly greater.

It was found that the solution of the fibrin in the stomach took place by means of hydrochloric acid, and that the same process could be imitated out of the body by mixing a quantity of hydrochloric acid, so small as scarcely to affect litmus paper, in distilled water, and immersing in it a morsel of fibrin. After twelve hours, at the the ordinary temperature, the fibrin was found converted into a gelatinous mass, which, if dissolved in distilled water and filtered, could not be recognized to differ in any chemical character from the fibrin found dissolved in the stomach of the dog. These experimentalists, therefore, regard the hydrochloric acid as the essential agent in the solution of the fibrin; and as the experiments of Hallé, Magendie, and other physiologists prove, that alcohol and other fluids, colored and uncolored, are removed directly from the stomach by means of the venous system, and cannot be recognized in the chyle while their presence can be detected in the blood, Bouchardat and Sandras have no hesitation in stating, as the result of their observation, that the same takes place with the fibrin after it has once become dissolved.

They performed analogous experiments on dogs with gluten, and found the very same results were arrived at. It formed a solution in the stomach precisely similar to that of fibrin; was removed from the stomach in the same manner, without passing into the intestines in any appreciable quantity; and dissolved out of the body by means of water slightly acidulated with hydrochloric acid. Albumen and pure caseum underwent solution in diluted hydrochloric acid in the same manner, and furnished the very same reactions with chemical agents.

A number of other experiments were made on the digestion of starch, sugar, &c., from which it appeared that these substances all became converted in the stomach into lactic acid, and were removed from it directly in the same manner as the dissolved fibrin, gluten, &c., had been. The chyle of animals fed with these non-azotized matters yielded no traces of starch and scarcely any of acid. In all animals the chyle was alkaline, or quite neutral. When dogs are fed on substances which they loathed, and would have vomited were the œsophagus not tied, the chyle was neutral, but when fed on bread or potatoes was always alkaline.

Experiments were also made on feeding dogs with fat. It was found that this substance was not altered in properties in the stomach, as pure fat was obtained from the pultaceous mass. The other liquids present in the stomach were acid, and contained hydrochloric acid. In the duodenum a yellowish-colored emulsive mixture was found, with a neutral reaction, which yielded fat when washed with ether. The other small intestines contained also a similar matter which furnished fat to ether. The thoracic duct furnished a white milky-looking chyle, much whiter than was ever seen in the former experiments, and when washed in ether furnished a notable quantity of fat.

These experiments seemed to prove that fat is digested in a very different way from the other nutritive matters; that it undergoes no change in the stomach, but that its chief changes occur in the duodenum; that, in fact, those changes occurred to it there which facilitated its absorption. These changes are, however, very simple. The pancreatic fluid and the bile mixing with the fatty matters, form a simple emulsion, without changing the chemical nature of the fatty matters. If these contain margaric and oleic acids in their natural state, they are saturated by the alkali contained in the pancreatic juice and in the bile. In this state they are absorbed by the orifices of the chyliferous vessels, and from thence carried into the thoracic duct, and mixed with the chyle. The analysis of the chyle proves this fact, and the examination of the contents of the bowels, at all parts of their course, prove that they contain fatty matters, which, if given in too large quantity, are even excreted with the feculent matters.

Bouchardat and Sandras draw the following important conclusions from their experiments:—

1. In digestion, the functions of the stomach consist in dissolving with the aid of hydrochloric acid, all albuminous matters, as fibrin, albumen, caseum, and gluten.

2. This acid, if diluted with 5000 parts of water, dissolves the same matters out of the body, provided they are not cooked; but if boiled the solution has no action on them. As they are found to be dissolved, however, in the stomach, it is probable that some other agent is at work than simple solution by means of hydrochloric acid: but the presence of that acid seems to be always indispensable.

3. As far as the albuminous matters are concerned, digestion and absorption take place exclusively in the stomach; the intestines present scarcely any traces of those dissolved matters which exist in such abundance in the stomach.

4. Solution of fecula also occurs in the stomach. This principle does not appear to pass into the state of sugar, and the experiments do not even warrant the statement that it passes into that of soluble starch; we regard as proved, its transformation into lactic acid.

5. The absorption of this kind of aliment seems to take place less exclusively from the stomach than that of the albuminous matters, a circumstance which would accord with the particular disposition and length of the intestines in animals not carnivorous.

6. Fatty matters are not attacked in the stomach. They pass into the duodenum in a state of emulsion, in consequence of the alkalies furnished by the liver and pancreas.

This emulsion is found abundantly throughout the whole course of the intestines.

7. The chyle has appeared to be somewhat less abundant, but presenting similar characters in the animals which were killed after long fasting, and in those killed after being fed on copious meals of albuminous matters and of fecula. It has only presented a marked difference in those fed with fat, when this principle was met with in it in considerable proportion.

Differing as these views do from those at present in vogue relative to digestion, Bouchardat and Sandras add a few remarks on the peculiarity of their views and the probable use of the chyliferous system of vessels. It is to be remembered that they found that the chyle procured during the digestion of fibrin did not differ in a single character from that procured from an animal fed on fecula alone, or in a state of starvation. That, in fact, these matters had not been converted into chyle. One important fact they ascertained to be was, that these chyliferous vessels absorbed the fatty matter, but this cannot be their sole use. During digestion a large quantity of hydrochloric and lactic acids are secreted and thrown into the stomach. These acids must come from the decomposition of salts existing in the system—chloride of sodium and lactate of soda. The abdominal glands prepare for the chyliferous vessels and thoracic canal, a chyle, the alkalininity of which is greater in proportion to the acidity developed in the stomach; and thus chyle, which is not solely produced by the absorption of aliments, but by a process of true secretion, mixes with the blood, to neutralize the acid which was indispensable for the solution of the food in the stomach. This simple process allows the blood to be continually repaired, without appreciably changing its nature.—*Annales des Sciences Naturelles*, October, 1842. *Bulletin Med. Sci.*

